# Multimorbidity in dementia: Current perspectives and future challenges

**DOI:** 10.1002/alz.70546

**Published:** 2025-08-04

**Authors:** Lucy E. Stirland, Radmila Choate, Preeti Pushpalata Zanwar, Panpan Zhang, Tamlyn J Watermeyer, Martina Valletta, Mario Torso, Stefano Tamburin, Usman Saeed, Gerard R. Ridgway, Shirine Moukaled, Jay B. Lusk, Samantha M. Loi, Thomas J. Littlejohns, Elżbieta Kuźma, Sarah‐Naomi James, Giulia Grande, Isabelle F. Foote, Katheryn A. Q. Cousins, Joe Butler, Abrar AbuHamdia, Thiago J. Avelino‐Silva, Vidyani Suryadevara

**Affiliations:** ^1^ Division of Psychiatry Institute of Neuroscience and Cardiovascular Research University of Edinburgh Edinburgh UK; ^2^ Global Brain Health Institute University of California San Francisco Memory and Aging Center San Francisco California USA; ^3^ Department of Epidemiology and Environmental Health College of Public Health University of Kentucky Lexington Kentucky USA; ^4^ Sanders‐Brown Center on Aging University of Kentucky Lexington Kentucky USA; ^5^ Irma Lerma Rangel College of Pharmacy Texas A&M University Kingsville Texas USA; ^6^ Department of Biostatistics Vanderbilt University Medical Center Nashville Tennessee USA; ^7^ Vanderbilt Memory & Alzheimer's Center Vanderbilt University Medical Center Nashville Tennessee USA; ^8^ Department of Psychology Faculty of Health & Life Sciences Northumbria University Newcastle‐Upon‐Tyne UK; ^9^ Edinburgh Dementia Prevention Centre for Clinical Brain Sciences University of Edinburgh Edinburgh UK; ^10^ Aging Research Center Department of Neurobiology Care Sciences and Society Karolinska Institutet Solna Sweden; ^11^ Oxford Brain Diagnostics Ltd Oxford UK; ^12^ Department of Neurosciences Biomedicine and Movement Sciences GB Rossi Hospital University of Verona Verona Italy; ^13^ Institute of Medical Science Temerty Faculty of Medicine University of Toronto Toronto Ontario Canada; ^14^ Hurvitz Brain Sciences Program Sunnybrook Research Institute Toronto Ontario Canada; ^15^ Department of Epidemiology Tulane University New Orleans Louisiana USA; ^16^ Department of Family Medicine University of North Carolina‐Chapel Hill Chapel Hill North Carolina USA; ^17^ Duke‐UNC Alzheimer's Disease Research Center University of North Carolina‐Chapel Hill Chapel Hill North Carolina USA; ^18^ Department of Psychiatry and Neuropsychiatry Centre University of Melbourne and Royal Melbourne Hospital Parkville Victoria Australia; ^19^ Nuffield Department of Population Health Big Data Institute University of Oxford Oxford UK; ^20^ Albertinen Krankenhaus/Albertinen Haus gGmbH Academic Teaching Hospital of the Faculty of Medicine University of Hamburg Hamburg Germany; ^21^ MRC Unit for Lifelong Health and Ageing University College London London UK; ^22^ Institute for Behavioral Genetics University of Colorado Boulder Boulder Colorado USA; ^23^ Department of Neurology University of Pennsylvania Philadelphia Pennsylvania USA; ^24^ School of Psychology University of Sunderland Sunderland UK; ^25^ Division of Genomics and Precision Medicine College of Health and Life Sciences Hamad Bin Khalifa University Doha Qatar; ^26^ Division of Geriatrics San Francisco Veterans Affairs Medical Center University of California San Francisco San Francisco California USA; ^27^ Laboratorio de Investigacao Medica em Envelhecimento (LIM‐66) Servico de Geriatria Setor Azul (Clinica Medica) Hospital das Clinicas da Faculdade de Medicina da Universidade de Sao Paulo Sao Paulo Brazil; ^28^ Department of Radiology Molecular Imaging Program at Stanford (MIPS) Stanford University School of Medicine Stanford California USA

**Keywords:** all‐cause dementia, comorbidity, multimorbidity, multiple long‐term conditions

## Abstract

Multimorbidity—the co‐occurrence of two or more chronic health conditions—affects > 86% of people with dementia. It is associated with cognitive and functional decline, reduced health‐related quality of life, increased health‐care use, and higher mortality. The relationship between multimorbidity and dementia is potentially bidirectional; conditions such as hypertension and diabetes increase the risk of developing dementia, and cognitive impairment can complicate their management. This complexity presents challenges in health care and research, affecting treatment decisions and often leading to the exclusion of these individuals from clinical trials. Understanding multimorbidity through long‐term prospective studies is crucial to clarify its relationship with dementia. Investigating specific disease combinations, environmental and genetic factors, and their impacts on cognitive health will guide the development of effective prediction models and inclusive intervention strategies for diverse global populations across the life course.

**Highlights:**

- Multimorbidity affects > 86% of individuals with dementia, worsening outcomes.
- The relationship between multimorbidity and dementia is potentially bidirectional.
- Chronic conditions hinder dementia management and clinical trial inclusion.
- Life‐course multimorbidity research is key to dementia risk reduction strategies.
- Prospective studies are needed to improve prediction models and interventions.

## Graphical abstract

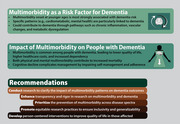

## 1 INTRODUCTION

As global populations age, both dementia and multimorbidity are gaining attention as priorities for research and public health. Dementia is a clinical syndrome characterized by cognitive, psychological, behavioral, and functional impairments.^1^ The most common causes of dementia include Alzheimer's disease (AD), vascular disease, Lewy body spectrum disorders, frontotemporal lobar degeneration, and mixed pathologies.^2^

### 1.1 Defining comorbidity and multimorbidity

Multimorbidity is usually defined as the co‐existence of two or more chronic health conditions in one person.^3^, ^4^ Some definitions specify that these can comprise long‐term physical diseases as well as mental health conditions, which in certain diagnostic systems include dementia.^1^, ^3^, ^4^, ^5^ When physical conditions are considered alone, this can be termed physical or somatic multimorbidity, and when both physical and mental conditions co‐exist, mental–physical (or somatic–mental) multimorbidity may be used.^6^, ^7^ Other terminology, often preferred by patients and the public, includes multiple long‐term conditions and multiple chronic conditions. Because the usage of these terms differs internationally, we use the broader term “multimorbidity” throughout this paper for consistency.^8^

Comorbidity, however, is described as the presence of any disease that may interact with a primary condition of interest.^3^ Therefore, in the context of other conditions, dementia can be considered either one component of multimorbidity or the primary condition of interest (known as an index condition) in relation to comorbidities.

A meta‐analysis of epidemiological studies from both high‐ and low‐ and middle‐income countries (HICs and LMICs) reported a pooled prevalence of multimorbidity of 33.1%, which increased with age regardless of sex.^9^ Multimorbidity is associated with poorer health outcomes, including increased mortality, impaired activities of daily living, delirium, reduced quality of life, and increased health‐care costs.^8^, ^10^ Due to the large number of possible combinations of conditions, people with multimorbidity often have unique phenotypes and may not be well served by specialty care that is oriented around a single diagnosis. Comorbidities are often part of exclusion criteria for clinical trial participation, leading to insufficient evidence to guide treatment decisions for people with multimorbidity.^11^, ^12^ In this era of increasing research and policy focus on the impact of both multimorbidity and the projected increase in dementia prevalence, this is a pertinent time to reflect on their relevance to each other and to consider ways to mitigate their future impacts.

### 1.2 Relevance of multimorbidity to dementia

Dementia is a significant health and societal issue and a leading cause of death worldwide. People with dementia commonly have multiple comorbidities, with one study of nearly 150,000 primary care patients reporting that 86.7% of people with dementia had at least two other conditions, compared to 63.9% of matched controls without dementia, and that 74.8% of people with dementia had three or more different conditions, compared to 53% of controls.^13^ Other research has reported that people with dementia have a mean of four comorbidities, compared to two in those without dementia,^14^, ^15^ and that people with dementia alongside six or more chronic conditions have particularly poor health.^16^ The combination of chronic conditions in an older adult with dementia adds complexity due to interactions between comorbidities and the use of multiple prescribed medications, increasing the risk of adverse health outcomes.

Due to the heterogeneity of multimorbidity phenotypes, there are likely to be multiple mechanisms underpinning its association with dementia. These may include causative relationships between certain conditions and neurodegeneration, common risk factors for multimorbidity and dementia, or the impact of specific conditions or treatments on cognitive reserve and function. In turn, the variety of clinical manifestations and neuropathological presentations of dementia represents complex pathways between systemic conditions and causes of neurodegeneration. For example, hypertension, diabetes, and heart disease are often comorbid with vascular dementia, and the presence of these conditions may influence the clinical diagnostic process. Furthermore, diagnosing specific subtypes of dementia, especially when cognitive impairment occurs in the context of multimorbidity, can be difficult and is often impractical in many settings, particularly in LMICs.

The interplay between multimorbidity and all‐cause dementia may be bidirectional; not only is there an established link between multimorbidity and developing dementia,^17^ but the presence of cognitive impairment can impair the optimal management of other comorbidities or risk factors, thereby contributing to a greater burden of multimorbidity and impeding dementia risk reduction strategies.^18^ Balancing the prioritization of dementia or other conditions poses challenges in implementing person‐centered care and for health‐care systems.^19^ Recognizing that dementia usually presents within a complex clinical context is essential for providing comprehensive care.

**FIGURE 1:**
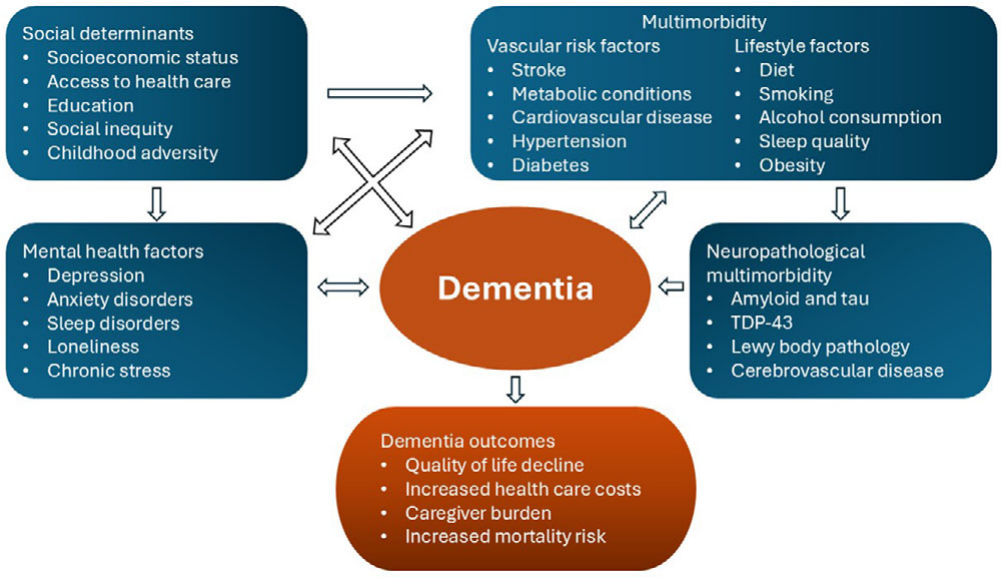
Flowchart of interactions, common causes, and consequences of co‐existent multimorbidity and dementia. TDP‐43, transactive response DNA‐binding protein 43 kDa.

Figure 1 summarizes the interactions, common causes, and consequences of co‐existent multimorbidity and dementia.

### 1.3 Neuropathological multimorbidity

Although the concept of multimorbidity encompasses conditions across body systems, the co‐existence of multiple pathologies within the brain is also common, and systemic conditions may influence the accumulation of specific pathologies in numerous ways.^20^ AD is neuropathologically characterized by the presence of misfolded amyloid beta (Aβ) and tau proteins, although there is increasing evidence of co‐pathologies including α‐synuclein,^21^ transactive response DNA‐binding protein 43 kDa (TDP‐43),^22^ and cerebrovascular disease.^23^ The relevance of this comorbid neuropathology is acknowledged by the 2024 revised criteria for the diagnosis and staging of AD.^24^ These pathologies often co‐occur to varying degrees as “mixed” disease, are more common than single “pure” pathologies,^25^ and are associated with more severe dementia and faster disease progression.^26^

It is unknown how physical conditions affect the risk for multiple neurological pathologies. An autopsy analysis of 767 brain tissue donors concluded that physical multimorbidity was not associated with dementia‐related neuropathological changes; instead, conditions that may be clinical or prodromal manifestations of dementia‐related neuropathology (Parkinson's disease, cerebrovascular disease, depression, and other psychiatric conditions) better predicted dementia‐related neuropathology at autopsy.^20^ This poses uncertainties about the role of comorbidities in increasing neuropathology, although a floor effect could take place in end‐stage disease. Vascular risk factors are strongly linked to markers of cerebrovascular disease (such as white matter hyperintensities), suggesting a complex relationship between multiple risk factors.

The resistance versus resilience framework is an alternative approach to understanding the co‐existence of multiple neuropathologies.^27^ Resistance refers to high‐risk individuals not developing neuropathology as expected, and resilience is defined as those with evidence of neuropathological damage who do not manifest clinical dementia. In research that focuses on single neuropathological processes, apparent resilience to one cause of dementia may in fact represent resistance to another comorbid pathology that was not under study.^28^

## 2 EPIDEMIOLOGY THROUGH THE LIFE COURSE

### 2.1 Methodological challenges

In the last decade, as research and policy interest in multimorbidity has grown, there has been an influx of evidence on the associations between multimorbidity and dementia, with two 2025 systematic reviews capturing their characteristics.^17^, ^29^ The complexity of multimorbidity is reflected in the various methods of measuring it, with inconsistencies affecting prevalence estimates and reported associations with relevant outcomes. A 2021 systematic review on multimorbidity found that only 47.3% of studies used the accepted definition of two or more co‐existing conditions, with more than one third of the papers not defining their threshold at all.^6^ The review also highlighted the importance of determining how many and which candidate conditions were included (this ranged from 2 to 285) and how these were grouped (e.g., counting myocardial infarction and angina as two separate conditions or grouping them into coronary heart disease). Because most multimorbidity studies have been designed to capture a range of diseases without reference to an index condition, dementia may appear as one contributor among a list of conditions. Therefore, when examining dementia alongside comorbidities, there is a risk of double‐counting if dementia itself, or related neurodegenerative disorders—for example, Parkinson's disease or stroke—appear among the included conditions.^20^, ^30^

Although two thirds of the 566 studies in the 2021 systematic review measured multimorbidity using counts of conditions, 155 (27%) studies used weighted indices, most commonly the Charlson Comorbidity Index, which assigns weights to each condition according to its association with mortality.^31^ Decisions about whether to use counts or indices depend on the study's aim and the data available.^32^ Disease counts are considered most appropriate when estimating prevalence, studying clusters of conditions, or measuring trajectories of multimorbidity accumulation, and indices are better suited for risk adjustment, assessing the severity of disease burden, or predicting the specific outcome for which the index was designed.^33^

For both multimorbidity and dementia ascertainment, attention should be paid to the sources of diagnostic data, such as self‐report (present in 55% of multimorbidity studies), extraction from health‐care records, or expert clinical diagnoses. Each of these approaches has its distinct limitations that may bias the results.^6^ A combination of methods may be used to explore multimorbidity and dementia together, such as clinical dementia diagnosis alongside multimorbidity ascertained from health records.

A further methodological challenge is the consideration of age. Multimorbidity research often focuses on older people, although its prevalence is high at younger ages in socioeconomically deprived groups.^4^ Although studying older adults captures most people with dementia, age limits in epidemiological studies of multimorbidity may exclude people with young‐onset dementia (often defined as onset before 65 years).

When examining the epidemiology of multimorbidity and dementia, questions arise about causation and correlation. To support a causative role, evidence should show that comorbid conditions pre‐date dementia. Due to the long prodromal phase of dementia, longitudinal studies of ≥ 10 years are likely necessary to account for the substantial impact of reverse causation driving observed associations. It may be that some apparently comorbid conditions diagnosed before dementia, for example depression, are in fact early manifestations of the dementia and are misrepresented in epidemiological studies as independent risk factors. Most longitudinal studies on multimorbidity and dementia to date have examined relationships between condition counts and incident dementia,^17^, ^29^ with consideration of the accumulation of specific conditions over time less well studied.^34^, ^35^

### 2.2 Importance of age with multimorbidity on dementia risk

As populations age, the prevalence of both multimorbidity and dementia is increasing.^36^ Emerging research suggests a link between multimorbidity in mid‐life (the prevalence of which is reported as 33% to 72% ^37^, ^38^, ^39^) and dementia.

Existing evidence is largely based on participants with multimorbidity measured at late mid‐life (≥ 60 years) or late life (≥ 70 years). The 2024 Lancet Commission on Dementia positioned individual risk factors within a life‐course framework, proposing that, among other conditions, hypertension, hearing loss, diabetes, and depression in mid‐life, and visual loss in late life, increase dementia risk.^40^ Addressing these conditions may reduce the risk of both dementia and comorbidities in later life.

Among 10,308 UK‐based participants from the Whitehall II study, multimorbidity onset at ages 55, 60, 65, and 70 years was associated with increased dementia incidence, with the strongest associations observed at younger ages, which progressively attenuated at older ages.^41^ For example, at age 65 years, participants with multimorbidity onset before age 55 (duration over 10 years) had a hazard ratio of 2.46 for subsequent dementia, compared to 1.51 for those with onset between 60 and 65 years.

Similar findings were observed among 23,196 participants of the Survey of Health, Ageing, and Retirement in Europe, in which multimorbidity was associated with increased incidence of dementia over 15 years of follow‐up, particularly in people aged < 55 years.^42^ Longitudinal studies like this provide additional knowledge toward understanding causality beyond what is shown in cross‐sectional research.

Multimorbidity in mid‐life might be a particularly strong risk factor for dementia due to the cumulative effects of protracted exposure. Understanding the accumulation of conditions over the life course can provide insights into whether there are critical risk periods and cumulative effects. Among 5923 participants of the US Health and Retirement Survey, rapid onset of conditions over time, but not slow or steady onset, was associated with increased dementia incidence.^35^

Future longitudinal studies should assess the relationship between multimorbidity across different life stages and dementia in later life, considering nuances such as the number, type, severity, patterns, and rate of accumulation of chronic conditions, and their relationship with different dementia subtypes. This precision evidence would inform intervention strategies to prevent or delay both multimorbidity and dementia at the earliest opportunity.

### 2.3 Multimorbidity patterns

Multimorbidity is heterogeneous, but its association with dementia might only be driven by the presence of certain conditions. Several studies have used the UK Biobank cohort of half a million participants to identify multimorbidity patterns. Within each study, a “cardiometabolic,”^43^ a “mental health,”^44^ and a broadly defined multi‐system multimorbidity pattern^45^ displayed the strongest associations with dementia. These variable results are likely due to the use of different methodological approaches to identify patterns. However, vascular and metabolic conditions were consistently related with dementia. Among 2478 participants from the Swedish National Study on Aging and Care in Kungsholmen, individuals with “neuropsychiatric,” “cardiovascular,” and “sensory impairment/cancer” patterns had higher rates of dementia.^46^ Understanding the role of patterns is important for identifying individuals at a higher risk of dementia and for the development of targeted preventative interventions. In addition, most epidemiological evidence to date is from HICs, and further investigation in a greater diversity of settings is a crucial next step.

### 2.4 Common risk factors for multimorbidity and dementia

As certain conditions, such as hypertension and diabetes, are established risk factors for all‐cause dementia, their co‐existence or interaction with other conditions may have an additive effect on dementia risk.^40^ Vascular risk factors such as smoking and hypercholesterolemia have been associated with the development of both AD and vascular dementia,^47^ highlighting that optimization of cerebrovascular risk factors could reduce dementia incidence through multiple mechanisms, as well as improve overall health.

Multimorbidity composition may vary in HICs and LMICs with different contributing conditions, although some demographic, socioeconomic, and lifestyle factors are common to all settings (for example, physical activity as a protective factor).^48^ In HICs, socioeconomic status and inequalities are linked with higher prevalence of multimorbidity, frailty, disability, and dementia,^49^, ^50^ suggesting a complex interplay of all these factors. Comprehensive investigations of multimorbidity risk in LMICs are lacking, but existing evidence highlights the role of socioeconomic status, social inequities, childhood adversity, lifestyle behaviors, obesity, and dyslipidemia.^51^

The impact of shared genetic pathways between risk factors for multimorbidity and dementia has been less explored. However, a genomic structural equation modeling study demonstrated a high level of genetic overlap between known modifiable risk factors for dementia.^52^ A twin study suggests that cardiometabolic multimorbidity, particularly in mid‐life, is associated with an increased incidence of dementia and that a genetic background underpins this association.^53^ In another UK Biobank study, there were stronger associations between multimorbidity and dementia in participants with a lower genetic risk of dementia (defined as non‐carriers of apolipoprotein E ε4). In those with high genetic predisposition, the direction of associations remained similar, albeit weaker in strength.^43^ Mendelian randomization studies in a multivariable framework might help researchers understand the role of genetic risk factors for multimorbidity and dementia and the mediating effects of environmental, socioeconomic, and lifestyle factors.^54^

Several studies have highlighted the role of multimorbidity in mild cognitive impairment (MCI). A longitudinal study and a cross‐sectional study of older adults in HICs reported the risk of MCI in people with multimorbidity to increase by 1.38 and 3.03 times, respectively, ^55^, ^56^ with a study of adults across six LMICs finding the risk increased by 1.40 times.^57^ Variations in the strength of association between studies could be related to participants’ disease profiles, sample age, sex, quality and accessibility of health care, poverty, and education levels.

### 2.5 Mental illness within multimorbidity

Most multimorbidity measures include mental illnesses. These disorders have a complex relationship with dementia; they may manifest as an early symptom of dementia, exist as a separate condition alongside dementia and additively reduce brain reserve, or act as a risk factor for dementia. Notably, meta‐analyses demonstrate that people with depression have a higher risk of developing dementia in later life compared to those without,^58^, ^59^ and, albeit less well studied, anxiety disorders have also been associated with cognitive decline and subsequent dementia.^60^ Additionally, depression has been documented as a risk factor for mortality in people with dementia.^61^ Mental illnesses may affect other health behaviors such as physical activity, smoking, and alcohol use,^62^ which may influence the progression of dementia.^63^ Addressing mental health, psychosocial factors, and related health behaviors in multimorbidity may mitigate adverse outcomes.

### 2.6 Potential causative pathways

The heterogeneity of multimorbidity means that there are multiple potential causative pathways toward dementia. Inflammation has been proposed as a primary mechanism for their interaction and is being explored as a potential target for dementia treatments.^46^, ^64^ Both alone and in combination, chronic conditions are associated with increased inflammatory cytokines, such as interleukins, tumor necrosis factor alpha, and plasminogen activator inhibitor‐1.^65^ Microglial inflammation is posited to occur concurrently with or after systemic symptoms appear.^66^

Normal cognitive function depends on the efficiency of cerebral blood perfusion and neuronal viability, as well as the availability of glucose, the primary fuel for the brain. Other mechanisms affecting the relationship between chronic diseases and cognition include hormonal alterations, such as insulin resistance or fluctuating blood glucose; reduced insulin‐like growth factor 1 in non‐alcoholic fatty liver disease; vitamin and protein deficiencies, such as decreased vitamin B12 and folate and increased homocysteine levels; autonomic dysfunction; fat accumulation in tissues and blood vessels^67^; and immune system activation.^68^, ^69^ Preclinical investigations of multimorbidity mechanisms have focused on the common vascular pathways with neurodegeneration.^70^ The gut microbiome has also been identified as a potential regulator of AD pathology and may be influenced by other factors, including genetics, lifestyle, and drugs.^71^ Although less biologically certain, there is evidence of a bidirectional association between medication use and functional impairment.^18^ People with multiple conditions usually take several medications (known as polypharmacy), and this has been identified as associated with dementia, with potential causation through anticholinergic burden.^72^

## 3 IMPACT OF COMORBIDITIES ON PEOPLE WITH ESTABLISHED DEMENTIA

Multimorbidity is associated with poorer physical, mental, and social health outcomes and reduced independence.^73^, ^74^ This decline in health‐related quality of life (HRQoL) is exacerbated in individuals with dementia, as both cognitive and physical health deteriorate simultaneously.^75^ Additionally, people with dementia and multimorbidity often experience social isolation, which can lead to loneliness, mobility problems, pain, mood disturbances, and challenges in managing daily activities.^16^, ^75^, ^76^ Certain comorbid conditions, such as genitourinary, sight, or oral health problems, are associated with substantially diminished HRQoL and affected social interactions.^16^ Accurate evaluation of HRQoL in people with dementia can be influenced by reliance on self‐assessment of health status and the potential bias introduced by proxy reporting from caregivers.^16^

Increased comorbidity contributes to mortality in dementia beyond the impact of dementia alone.^77^, ^78^, ^79^ This may be explained by under‐treatment of comorbidities, impaired self‐management and decision making, and challenges with following recommended treatment plans.^80^, ^81^, ^82^

### 3.1 Person‐centered perspectives

Managing both dementia and comorbidities can be complicated. There is little guidance for clinicians on how to optimize prescribing for this population, and several barriers exist to shared decision making about taking multiple medications.^83^ Deprescribing might reduce the risk of adverse events but requires culturally sensitive communication within a trusted patient–physician relationship.^84^

Data from the National Health and Aging Trends Study and the National Study of Caregiving show that among people with dementia, each additional comorbidity adds physical and psychological demands on caregivers.^85^ Qualitative research on the experiences of people with multimorbidity has shown the importance of a person‐centered and family‐centered approach to community care. This includes coordination of health and social services tailored to the needs of older adults and their informal caregivers.^86^ Another study on the care needs of nursing home residents showed differences between the perspectives of residents with mental and physical comorbidity and nursing staff.^87^ It recommended developing a dialogue about needs, wishes, and expectations to optimize individually tailored care plans.

People with dementia and multimorbidity require multidisciplinary teams to address their complex needs. Care strategies should focus on managing comorbidities and may benefit from care coordination around hospital admissions, caregiving, and health‐care provision, particularly in advanced dementia.

### 3.2 Health‐care use and costs

Multimorbidity research has shown that as the number of chronic conditions increases, so do health‐care use and costs.^88^ This association varies by country and increases with national gross domestic product, and most evidence comes from the United States.^89^ For example, a longitudinal study of older adults with multimorbidity and dementia estimated the total societal cost per beneficiary at US$44,786 in 2011, with the majority of costs arising from hospital admissions for physical illnesses.^90^

Dementia itself is associated with increased health‐care use and costs.^91^, ^92^ In the United States, Medicare costs escalate with the severity of dementia and increase further with higher numbers of comorbidities, with annual expenditures between 1999 and 2010 ranging from $2612 for no comorbidities to $30,244 for three or more comorbidities.^93^ Matching people with dementia to controls without dementia found a higher mean per‐person cost for each of the 15 comorbidities studied.^94^ Population‐based data from Hong Kong between 2010 and 2019 corroborated that an increasing number of comorbidities alongside dementia was associated with increased emergency department attendances and hospital admissions.^95^

Although dementia can be a terminal disease, with survival post‐diagnosis ranging between 1.8 and 7.2 years^96^ and post‐symptom onset ≈ 6.3 years,^97^ health‐care costs may decrease as the disease progresses. Studies from Sweden^98^ and Denmark^99^ reported higher costs related to dementia for up to 4 and 5 years, respectively, out of 10 years of follow‐up. This may reflect the fact that the focus of care often moves from medical intervention to assistance with activities of daily living as dementia progresses.^100^

## 4 CHALLENGES IN EMERGING CLINICAL DEVELOPMENTS

### 4.1 Relevance of multimorbidity to dementia clinical trials

People with chronic conditions are often excluded from drug trials.^101^ This is particularly relevant in the era of new disease‐modifying treatments for dementia, in which clinical trials often under‐report comorbidities.^102^ In 2022, the US Food and Drug Administration (FDA) granted accelerated approval of two monoclonal antibodies, aducanumab and lecanemab, the latter subsequently receiving traditional approval.^103^ Notable exclusion criteria for both drugs’ phase 3 trials included other neurodegenerative conditions or brain disorders, cardiovascular disease, cardiopulmonary contraindications, and a history of cancers, all of which are common in older adults. A simulation study applying these criteria to people with early‐stage AD in a population‐based cohort found that only 5% and 8% of their participants (*N* = 237) met eligibility for aducanumab and lecanemab, respectively.^104^

Similarly, a study applying aducanumab clinical trial criteria to a retrospective database from a specialist cognitive service found that although 57% of patients met appropriate use criteria for aducanumab, only 27% met clinical trial inclusion criteria.^105^ In comparison, another study showed that the proportion of memory clinic patients who would be excluded was as high as 99%.^106^ Such findings expose the uncertainty surrounding the external validity of dementia therapies in current trial pathways. To reduce variability and safety risks, the stringent criteria in such trials compromise the representation of common comorbidities and generalizability, thereby creating a gap in risk–benefit information for broader patient populations. In addition, people with vascular comorbidities are at higher risk of significant adverse effects from these monoclonal antibodies, with implications for their health and increased costs associated with enhanced monitoring.^107^, ^108^

### 4.2 Impact of multimorbidity on dementia biomarkers

#### 4.2.1 Blood biomarkers

Another recent advance in the management of dementia is the development of blood biomarkers of AD.^109^ Their introduction into clinical practice will help refine diagnoses and may be used to provide biomarker evidence of AD pathology before prescribing monoclonal antibodies. However, two studies of cognitively unimpaired people have found that blood concentrations of these biomarkers vary with comorbidities, namely chronic kidney disease, dyslipidemia, hypertension, and diabetes.^110^, ^111^ Further results in people without dementia also suggest that levels of four biomarkers (phosphorylated tau 181, total tau, neurofilament light chain, and glial fibrillary acidic protein) increase with the number of comorbid chronic conditions.^112^ Given the high prevalence of comorbidities in people likely to undergo blood biomarker testing for AD, it will be crucial to consider these findings when interpreting test results.

#### 4.2.2 Neuroimaging biomarkers

Neuroimaging studies of people without dementia have revealed a link between multimorbidity and biomarkers of neurodegeneration and cerebrovascular pathology.^113^, ^114^, ^115^ Multimorbidity was associated with smaller brain volumes, including total brain tissue and hippocampal volume, lower cortical thickness, and 18F‐fluorodeoxyglucose positron emission tomography hypometabolism.^113^, ^114^, ^115^ These studies also reported an association between multimorbidity and markers of vascular pathology, such as white matter hyperintensities and cortical infarcts. No association was observed with imaging markers of amyloid deposition.^115^ Notably, the association with neuroimaging biomarkers appeared stronger when multimorbidity affected multiple body systems ^113^ or in the presence of specific multimorbidity patterns.^114^ Multimorbidity may therefore influence brain structure independently of neurodegenerative diseases.

## 5 GLOBAL EQUITY

Multimorbidity is context dependent, and rates differ among ethnic groups and between countries.^116^, ^117^ Disparities exist not only between HICs and LMICs but also within nations, often reflecting underlying social inequalities. Health equity is defined as the absence of unfair, avoidable, or remediable differences between groups of people, which will allow all individuals to attain their full potential for health and well‐being.^118^ Marginalized and socioeconomically disadvantaged communities, including those affected by structural racism, frequently experience a higher prevalence of both multimorbidity and dementia.^4^, ^119^ These disparities are often masked, as many national datasets do not provide sufficiently detailed data on different ethnic and socioeconomic groups, thereby hampering efforts to understand and address the challenges faced by these populations. For example, multimorbidity is common among older South Asian Indians,^120^ but in many US national datasets, data are aggregated for all Asian groups. Furthermore, race and ethnic categories are social rather than biological constructs, and their definitions may vary according to the geopolitical context of research.^121^ These differences mean that ethnic categories are often not transferable between countries. For example, the precise composition of who is included in the “Asian” category may differ between the United States and UK. There is, therefore, a need for disaggregated and longitudinal data on subgroups of minority populations in the United States and globally to examine the prevalence, incidence, and modifiers of multimorbidity and the relationship to other age‐related chronic conditions, including dementia.^122^

Given the projected rapid population aging in LMICs in the coming decades, preventing and managing chronic diseases is a global research and public health priority.^123^ Addressing inequity requires culturally sensitive, affordable, and accessible health‐care interventions tailored to the needs of diverse populations. Integrated policy and system‐level approaches to managing multimorbidity and dementia include care models that combine medical, social, and community resources, with skilled care coordinators overseeing both dementia and co‐existing conditions.^82^ Continuity of care is known to reduce hospitalizations and emergency visits,^124^ so enhanced training for community health‐care providers on managing multimorbidity is needed, fostering generalist skills alongside specialization.^125^ Finally, system‐level changes, such as expanding health services and promoting interprofessional cooperation, are necessary to improve care delivery.^126^ By prioritizing equity in research, health‐care delivery, and policy making, it will be possible to reduce health disparities and improve outcomes for individuals affected by these complex conditions.

## 6 PREVENTION AND PUBLIC HEALTH STRATEGIES

There is some evidence for a decline in dementia incidence rates in HICs, attributed to national policy changes like compulsory education and reduced smoking rates, as well as healthier lifestyle and improved management of cardiovascular conditions.^127^ Given the common risk factors for multimorbidity and dementia, prevention efforts at individual and population levels will benefit both brain health and overall physical health cost‐effectively.^128^ For example, trials of multi‐domain risk reduction programs for dementia have shown that interventions can improve not only cognition but also improve cardiovascular risk factors and reduce the accumulation of chronic conditions.^129^, ^130^

Multimorbidity and the interaction between physical health, mental disorders, and brain health should be considered in dementia prevention strategies at global and national policy levels (primary prevention), among people with identified risk factors for dementia (secondary prevention), and in the care of people who have already developed cognitive impairment (tertiary prevention).

## 7 FUTURE RECOMMENDATIONS

### 7.1 Recommendations for more equitable research

Research can be a powerful tool in addressing inequities in multimorbidity and dementia. Modifiable risk factors for dementia disproportionately affect LMICs, as well as marginalized and socially disadvantaged communities in HICs.^40^, ^131^, ^132^ Despite the greater potential for risk reduction in these communities, they are often under‐researched in dementia studies. Therefore, incorporating equity, diversity, and inclusion considerations and collaborating internationally is crucial for conducting rigorous, relevant, and impactful research that is generalizable to all population groups.^133^ Key research recommendations are shown in Box 1, including those with general reach and with specific reference to multimorbidity, highlighting the fact that measures to improve multimorbidity research and outcomes can be relevant and beneficial to other areas of health.^19^

BOX 1 **Equity, diversity, and inclusion (EDI) recommendations for multimorbidity and dementia^19^**

- Promoting, funding, and enabling equitable research by governing bodies and agencies.
- Ensuring equitable access to funding opportunities for all researchers and trainees.
- Recruiting and training a diverse workforce to effectively engage with under‐researched groups.
- Building and maintaining relationships with under‐researched groups to foster trust, establish research priorities, and facilitate longitudinal data collection.
- Integrating EDI considerations into multimorbidity research design and data analyses.
- Developing and testing interventions and multimorbidity‐related outcomes in diverse populations.

### 7.2 Recommendations for reporting methods

As the field of multimorbidity and dementia continues to grow, research should be designed to promote quality, consistency, and reproducibility.^134^ Studies should report their definition of multimorbidity, the use of a list or an index, and the candidate conditions, including whether multiple conditions are grouped under a larger category or assessed individually.^6^ As in any study of an index disease, the definition and ascertainment of dementia should be specified, acknowledging that prevalence and resulting associations may vary by the methods used.^135^ In most dementia studies or trials, the term comorbidity will be most appropriate to describe co‐existing conditions alongside dementia (that is, with dementia being the index condition). We recommend that studies explicitly include important operational definitions in their inclusion criteria. In keeping with open research practices, study reports should publish their definition and measurement strategy for both multimorbidity and dementia, including clinical code sets and mapping to classification systems such as International Classification of Diseases 10th Revision. Advances in computational power will continue to facilitate more sophisticated assessment of the accumulation of conditions over time, using machine learning or artificial intelligence techniques. Studies using artificial intelligence should report the description of the datasets (test and training), relevant algorithms used, performance metrics, and the rationale and relevance of the applied method as recommended by existing reporting guidelines.^136^

### 7.3 Areas of research need

There is growing evidence for the association between multimorbidity and dementia, but their interactions are complex, requiring further exploration. Prospective longitudinal studies with repeat measures over long follow‐up periods could focus on the effects of accumulation of specific combinations of conditions as well as environmental and genetic factors to improve evidence around risk and causation, which will in turn better inform prevention and policy changes.

Now that the scale of multimorbidity among people with dementia has been better elucidated, more work is needed to ensure that the clinical application of new biomarkers and treatments accurately accounts for interacting conditions. Clinical trials for dementia treatments should consider matching inclusion criteria more closely to the characteristics of the affected population.

Interventions for managing the combination of multimorbidity and dementia are limited, so there is a need for person‐centered study design in this area.^137^ In addition, the importance of including people with dementia when setting research priorities is increasingly acknowledged. It is especially relevant when considering dementia alongside multiple comorbidities in the current context of resource allocation decisions about costly new treatments.^138^ Recommendations for research and policy are summarized in Box 2.

BOX 2 **Key recommendations for advancing research on dementia and multimorbidity**

- Design prospective, longitudinal studies to evaluate how combinations of chronic conditions, along with genetic and environmental factors, influence dementia risk.
- Engage individuals with dementia in setting research priorities and developing person‐centered interventions.
- Ensure clinical trial inclusion criteria reflect real‐world combinations and interactions of chronic conditions in people with dementia.
- Standardize definitions and measurement approaches for both multimorbidity and dementia across studies.
- Account for methodological variability when interpreting prevalence estimates of dementia and multimorbidity.
- Explore interactions among physical, mental, and brain health.

## 8 CONCLUSION

Multimorbidity significantly impacts the development, progression, and management of dementia, presenting complex challenges for individuals, caregivers, health‐care providers, and systems worldwide. The bidirectional relationship between multimorbidity and dementia underscores the necessity of comprehensive and integrated approaches to care that address both cognitive and physical health needs. There is compelling evidence of epidemiological associations throughout the life course, indicating that multimorbidity not only increases the risk of developing dementia,^17^ but also exacerbates cognitive decline,^139^ reduces HRQoL,^75^ and leads to higher health‐care use and costs.^93^, ^94^, ^95^

Emerging research on biomarkers and neuropathology enhances our biological understanding of how multimorbidity interacts with dementia, which is crucial for the development and clinical application of new diagnostic tools and treatments.^20^, ^110^, ^111^, ^112^ However, excluding individuals with multimorbidity from clinical trials limits the generalizability of findings and the effectiveness of interventions in real‐world settings.^101^ Therefore, future research must prioritize inclusivity and representation, ensuring that study populations reflect the diversity of those affected by both multimorbidity and dementia.

Addressing disparities and promoting equity are essential components of this effort. Policies and system‐level changes should focus on implementing integrated care models, adapting clinical guidelines to the needs of patients with multimorbidity and dementia, enhancing continuity of care, and fostering patient‐centered approaches.^19^ Training and education for health‐care providers on the complexities of managing these co‐existing conditions are also imperative.^126^

Future efforts should also concentrate on longitudinal studies to investigate the complex interactions between specific combinations of chronic conditions and dementia, considering behavioral, cultural, social, environmental, and biological factors.^140^ Inclusive clinical trials and person‐centered interventions will help tailor treatments to individual needs and preferences, improving outcomes and quality of life for those affected.

Optimizing physical and brain health through prevention strategies and integrated care models can achieve synergistic benefits that extend beyond individual patients to communities and health‐care systems.^130^ Embracing an approach that prioritizes equity and inclusivity will not only enhance understanding of multimorbidity and dementia but also pave the way for effective interventions that mitigate the societal and economic burdens of these intertwined conditions.

## CONFLICT OF INTEREST STATEMENT

The authors declare the following competing interests: G.R.R. and M.T. are currently employed at a company, Oxford Brain Diagnostics Ltd. All other authors declare no competing interests. Author disclosures are available in the supporting information.

## CONSENT STATEMENT

This paper does not report any original research with human participants so consent was not necessary.

## Supporting information



Supporting Information
